# The Primary Cultivation of Oogonial Stem Cells from Black Rockfish (*Sebastes schlegelii*): Morphology and Transcriptome Landscape

**DOI:** 10.3390/ijms26146772

**Published:** 2025-07-15

**Authors:** Jingjing Zhang, Lei Lin, Shengyu Zhu, Yanming Zhang, Caichao Dong, Yu Yang, Yuyan Liu, Xuwen Cao, Yangbin He, Honglong Ji, Bo Meng, Qian Wang, Changwei Shao

**Affiliations:** 1 State Key Laboratory of Mariculture Biobreeding and Sustainable Goods, Yellow Sea Fisheries Research Institute, Chinese Academy of Fishery Sciences, Qingdao 266071, China; jingjingzhang0307@163.com (J.Z.); linleicaas@163.com (L.L.); zhushengyu0703@163.com (S.Z.); z13109849329@163.com (Y.Z.); bingo0635@163.com (C.D.); yyu13156168974@163.com (Y.Y.); liuyy@ysfri.ac.cn (Y.L.); caoxw@ysfri.ac.cn (X.C.); heyb1002@163.com (Y.H.); 18733601983@163.com (H.J.); mengbo2510@163.com (B.M.); 2 Laboratory for Marine Fisheries Science and Food Production Processes, Qingdao Marine Science and Technology Center, Qingdao 266237, China; 3 College of Fisheries and Life Science, Shanghai Ocean University, Shanghai 201306, China; 4 Hebei Key Laboratory of the Bohai Sea Fish Germplasm Resources Conservation and Utilization, Beidaihe Central Experiment Station, Chinese Academy of Fishery Sciences, Qinhuangdao 066100, China

**Keywords:** *Sebastes schlegelii*, oogonial stem cell, primary cultivation, cell metabolism, cell cycle

## Abstract

Black rockfish (*Sebastes schlegelii*) is a marine ovoviviparous teleost that exhibits significant sexual dimorphism, with females growing faster and reaching larger sizes than males. Establishing stable oogonial stem cells (OSCs) is critical for understanding germline stem cell dynamics and facilitating all-female breeding. In this study, we successfully isolated and cultured OSCs from *S. schlegelii* for 12 passages. These cells exhibited alkaline phosphatase activity, expressed germline marker genes (*ddx4*, *cdh1*, *klf4*), and maintained a diploid karyotype (2n *=* 48). Transcriptomic comparisons between early (P3) and late (P12) passages revealed significant metabolic dysfunction and cell cycle arrest in the late-passage cells. Specifically, the down-regulation of glutathione-related and glycolysis-related genes (*gstm3*, *gstt1*, *mgst3*, *gsta1*, *gsta4*, *gsto1*, *gapdh*) and key mitotic regulators (*cdk1*, *chk1*, *cdk4*, *e2f3*, *ccne2*, *ccnb1*) suggested that metabolic imbalance contributes to oxidative stress, resulting in cell cycle inhibition and eventual senescence. This study provides a marine fish model for investigating metabolism-cell cycle interactions in germline stem cells and lays the foundation for future applications in germ cell transplantation and all-female breeding.

## 1. Introduction

In marine aquaculture, the black rockfish (*Sebastes schlegelii*) is considered an economically valuable species [1]. In 2024, the annual production of *S. schlegelii* in China reached 20,000 tons, reflecting its significant role in the domestic aquaculture market. Due to the fast growth rate of female *S. schlegelii*, the development of all-female breeding lines has emerged as an effective strategy for enhancing aquaculture productivity [1,2,3,4]. Germline stem cells (GSCs), particularly oogonial stem cells (OSCs), have been investigated as a promising resource for reproductive biotechnology, such as germ cell transplantation (GCT). However, stable and long-term in vitro culture systems for OSCs in marine fish remain underdeveloped.

GCT is an advanced reproductive technique in which donor-derived germ cells are introduced into the allogeneic or xenogeneic recipients, subsequently colonize the recipient’s genital ridges, proliferate, and differentiate into functional gametes. This technique has powerful applications in shortening the time of fishes’ sexual maturity, sex control breeding, and the conservation of rare and endangere·d fish. This technique has been studied and applied in a variety of teleosts [5], including *Oncorhynchus mykiss* [6], *Paralichthys olivaceus* [7], *O. masou* [8], *Danio rerio* [9], etc. Thus, the all-female production of *S. schlegelii* may be achieved by this method. Recent studies have found that primordial germ cells (PGCs), spermatogonial stem cells (SSCs), and OSCs have reproductive stem cell functions and can be used as donor cells for GCT in fish [10,11]. However, the number of OSCs is relatively small, because they begin to undergo meiosis immediately after sex differentiation [12], which greatly limits their utilization as donors in GCT. Therefore, the continuous culture of OSCs in vitro is the key to improve GCT technology in *S. schlegelii* for the production of all-female offspring.

At present, studies on the in vitro culture of OSCs are rare, and only a few somatic ovarian cell lines have been established in marine fish, including *Cephalopholis sonnerati* [13], *Epinephelus coioides* [14], *Larimichthys crocea* [15], *Verasper moseri* [16], *Silurus meridionalis* [17], *Ictalurus punctatus* [18], etc. Moreover, it has been shown that with various culture conditions, the in vitro culture of *S. schlegelii* ovarian cells can only maintained to the second passage [19]. Consequently, the ability to maintain OSCs in vitro over extended passages would not only facilitate germline engineering but also provide a platform to study the intrinsic regulatory mechanisms governing stem cell self-renewal and senescence. Previous studies in mammalian and freshwater fish models have shown that cellular metabolism and the cell cycle are intimately linked with stem cell function [20,21]. However, little is known about these interactions in marine teleost.

Here, we reported the successful isolation and long-term in vitro culture of OSCs from *S.schlegelii*. We further used transcriptomic analysis to compare early and late passage cells, aiming to elucidate the molecular mechanisms associated with OSCs’ aging and decline in vitro culture. Our findings have offered insights into the function of metabolic and cell cycle networks in GSCs’ fate and suggested strategies to improve OSCs’ sustainability in vitro. Our results could facilitate the in vitro culture of OSCs in ovoviviparous teleost and laid a foundation for generating all-female *S. schlegelii* stocks.

## 2. Results

### 2.1. Establishment of a Culture System for Oogonial Stem Cells from S. schlegelii

**Figure 1:**
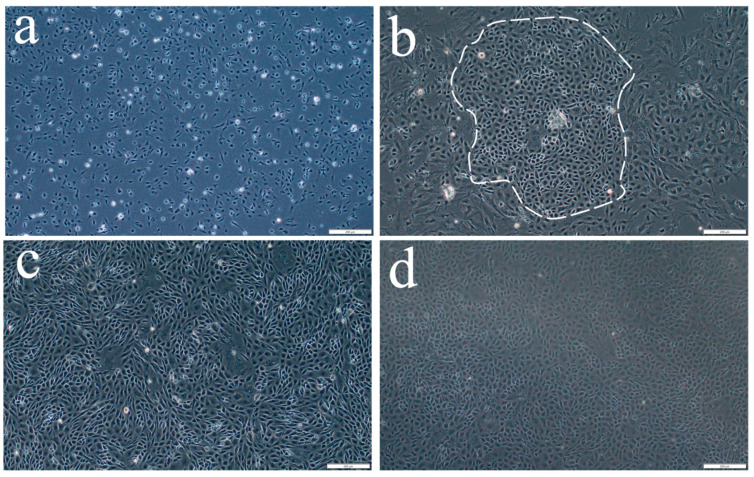
In vitro culture of *S. schlegelii* oogonial stem cells (SsOSCs). (**a**,**b**) Primary-cultured SsOSCs on the 1th and 7th days. The putative OSCs were circled. (**c**,**d**) Sub-cultured SsOSCs in passage 1 (19th day) and passage 10 (4th month). Scale bar = 200 μm.

**Figure A1:**
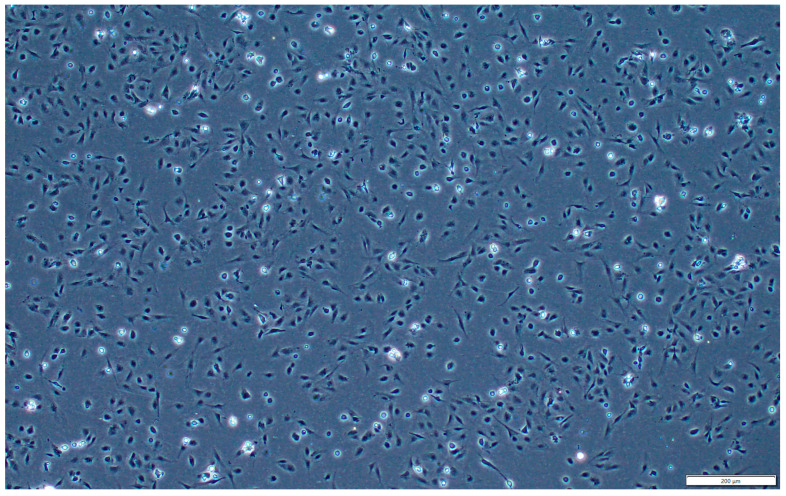
Subplot a of Figure 1. Primary-cultured SsOSCs on the 1st day. Scale bar = 200 μm.

**Figure A2:**
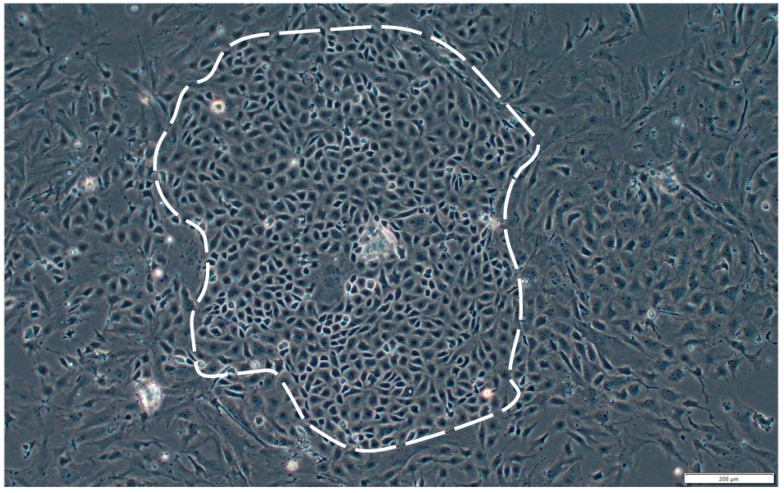
Subplot b of Figure 1. Primary-cultured SsOSCs on the 7th day. Scale bar = 200 μm.

**Figure A3:**
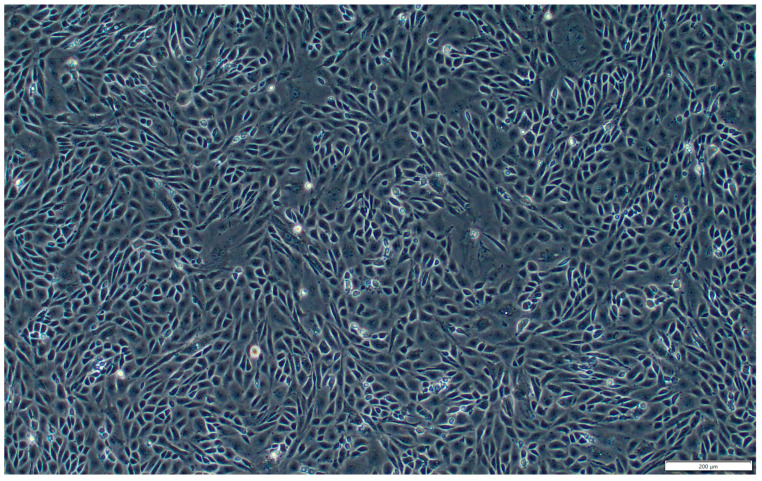
Subplot c of Figure 1. Sub-cultured SsOSCs in passage 1 (19th day). Scale bar = 200 μm.

**Figure A4:**
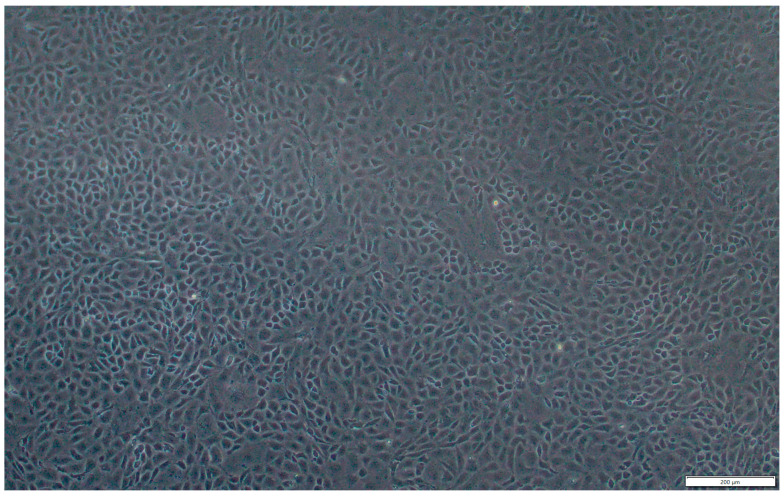
Subplot d of Figure 1. Sub-cultured SsOSCs in passage 10 (4th month). Scale bar = 200 μm.

OSCs isolated from the ovaries of one-year-old *S. schlegelii* were placed in culture flasks and cultured at 24 °C in complete medium. Following differential adhesion, the initially adherent cells were found to be fibrous (Figure 1a and Figure A1). Gradually the polygonal cells appeared to be adherent (Figure 1b and Figure A2), which were presumed to be the putative OSCs, and they were transferred to 96-well plates for monoclonal culture. After reaching approximately 90% confluence, they were digested and reseeded (Figure 1c,d, Figure A3 and Figure A4). The OSC cells in the passaged culture were polygonal and oval shaped.

### 2.2. Verification of Cell Origination

**Figure 2:**
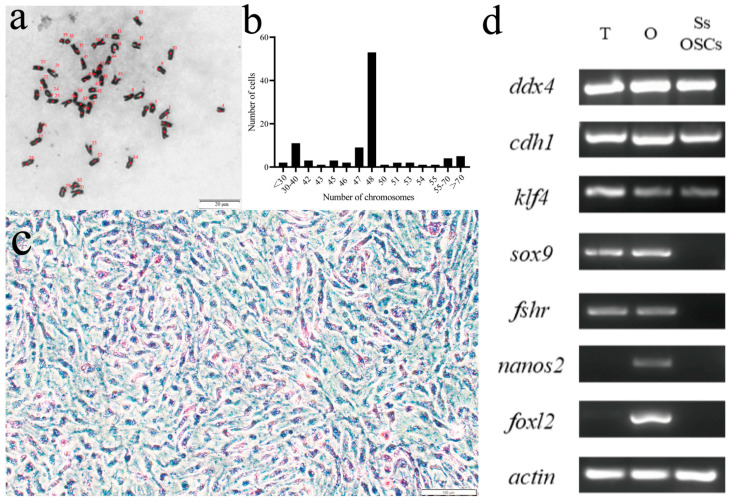
Verification of cell origination. (**a**) The chromosome morphology of SsOSCs. The numbers marked in red were the counts of chromosomes. Scale bar = 20 μm. (b) showed the chromosome number distribution of 100 cells. (**c**) The alkaline phosphatase staining of SsOSCs. Scale bar = 100 μm. (**d**) Expression of germ cell-related and somatic cell-related marker genes in testis (T), ovary (O), and SsOSCs.

**Figure A5:**
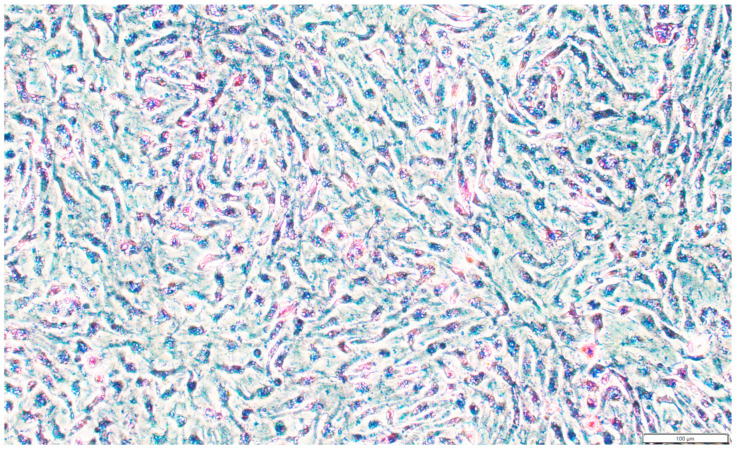
Subplot c of Figure 2. The alkaline phosphatase staining of SsOSCs. Scale bar = 100 μm.

We performed chromosome number counts on 100 cells in the dividing phase and found that the majority of cells maintained a chromosome number of 2n *=* 48 (Figure 2a), indicating that the chromosome morphology of SsOSCs was a diploid karyotype with 48 chromosomes (Figure 2b). Upon the establishment of stable SsOSCs, alkaline phosphatase staining demonstrated strong enzymatic activity in 70% of the adherent population (Figure 2c and Figure A5). The expression of germ cell-specific marker genes *ddx4* and *cdh1*, as well as the reproductive stem cell marker gene *klf4,* were detected. However, the somatic cell marker genes *sox9* and *fshr* were not expressed. Also, the marker genes of ovarian granular cells *nanos2* and *foxl2* were not expressed, indicating that these cells were not differentiated cells (Figure 2d).

**Figure 3:**
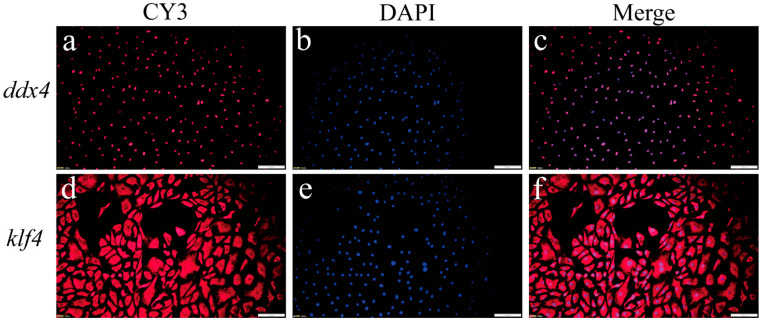
Detection of germ cell marker gene and stem cell marker gene in passage 12 (P12) SsOSCs. (**a**) The localization of *ddx4* in SsOSCs (red). (**b**) Cell nuclei were stained with DAPI (blue). (**c**) Merged image. (**d**) The localization of *klf4* in SsOSCs (red). (**e**) Cell nuclei were stained with DAPI (blue). (**f**) Merged image. Scale bar = 100 μm.

A germ cell-specific marker gene and a stem cell marker gene expression were further analyzed through fluorescence in situ hybridization. The majority of cells exhibited strong germ cell-specific marker gene *ddx4* signals (Figure 3a–c), with nearly universal stem cell marker gene *klf4* expression (Figure 3d–f). These findings confirmed the successful derivation of OSCs.

### 2.3. Morphological Changes in SsOSCs During In Vitro Culture

**Figure 4:**
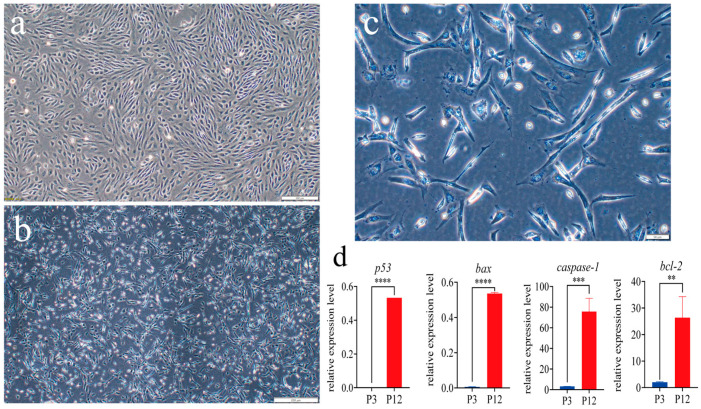
The morphology of different-passage SsOSCs. (**a**) Passage 3 (P3) cells at 10× magnification. Scale bar = 200 μm. (**b**) P12 cells at 10× magnification. Scale bar = 200 μm. (**c**) P12 cells at 40× magnification. Scale bar = 50 μm; (**d**) Relative expression level of apoptosis-related genes (*t*-test, ** *p* < 0.01; *** *p* < 0.001; **** *p* < 0.0001).

Most of the cells at passage 3 (P3) were oval or round (Figure 4a). The passage 12 (P12) cells changed, with the cell size increasing, an elongation of cytoplasm, and a flat, oblong polygonal shape. Furthermore, some of the P12 cells showed an inadequate membrane, enlarged nuclei, and instances of cell fusion. These morphological changes indicated that cell apoptosis may occur in the process of cell culture (Figure 4b,c). qRT-PCR analysis showed that P12 cells had a significant rise in the expression of apoptosis-related genes *p53* and *bax* (*p* < 0.0001), as well as *caspase-1* (*p* < 0.001) and *bcl-2* (*p* < 0.01), compared with P3 cells (Figure 4d).

### 2.4. Transcriptome Sequencing and Assembly Analysis

In order to explore the reasons that *S. schlegelii* OSCs cannot be cultured in vitro sustainably, we performed transcriptome analysis on P3 and P12 cells. Six cDNA libraries (termed P3_1, P3_2, P3_3, P12_1, P12_2, P12_3) were constructed. A total of 278,446,674 raw reads were generated, followed by quality control, leaving 262,860,298 clean reads. The average of Q20 and Q30 were 98.45% and 95.74%, respectively. Subsequently, the clean data were mapped to the *S. schlegelii* genome. Finally, 94.40% of the clean reads were mapped to the *S. schlegelii* genome (Table 1). The exceptional sequencing quality of these data guarantees the dependability of the subsequent transcriptome analysis findings.

**Figure 5:**
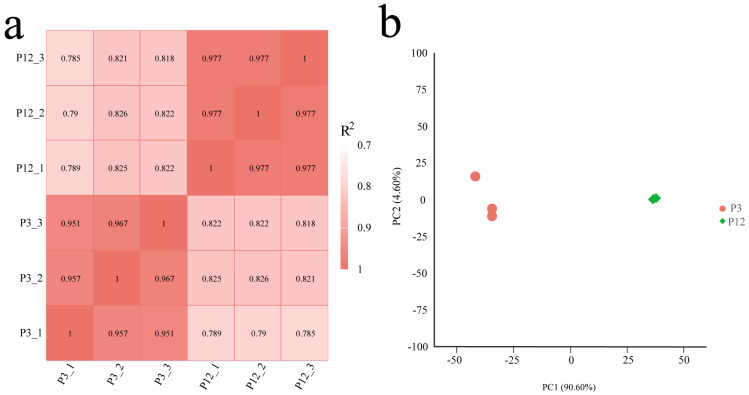
Correlation analysis of samples used in this study. (**a**) Pearson’s correlation coefficients between different samples. R^2^ represented the square of Pearson’s correlation coefficient. (**b**) Principal component analysis (PCA) of samples.

Based on the FPKM (Fragments Per Kilobase of exon model per Million mapped fragments) values of all the genes in each sample, the correlation coefficients of the intra- and inter-group samples and principal component analysis (PCA) were calculated. The results showed that the differences among the three biological replicates of each group were small, and the correlation coefficient was high (R^2^ > 0.951, Figure 5a). In addition, the two experimental groups could be clearly distinguished by PC1, indicating that these data could be used for further analysis (Figure 5b).

**Figure A6:**
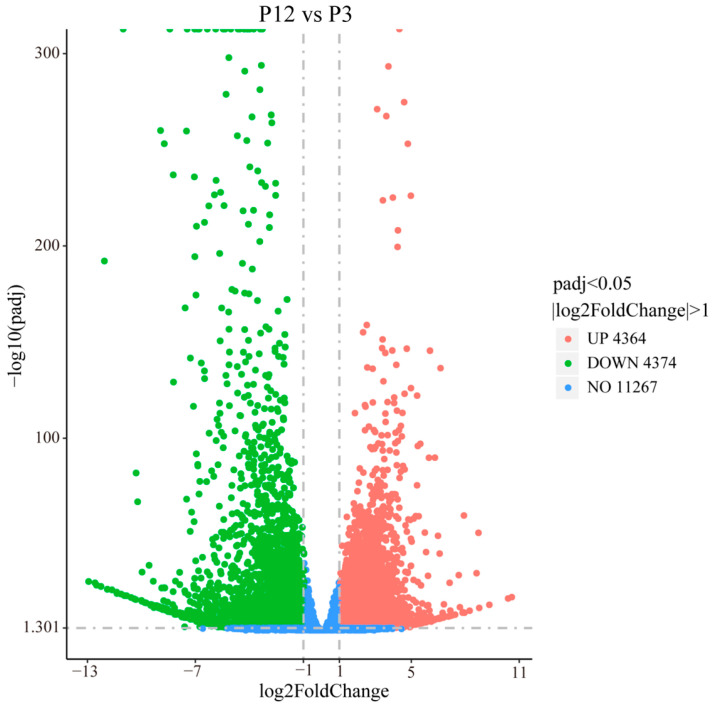
The volcano plot shows the distribution of gene expression levels. Green dots indicate down-regulated gene expression levels, red dots indicate up-regulated gene expression levels, and blue dots indicate genes that were not significantly different.

Volcano plot analysis was employed to depict the differentially expressed genes (DEGs). A total of 8738 DEGs were identified between the two groups, with 4364 significantly up-regulated and 4374 significantly down-regulated in the P12 group compared to the P3 group (Figure A6, Table S1).

### 2.5. GO and KEGG Enrichment Analysis of DEGs

**Figure 6:**
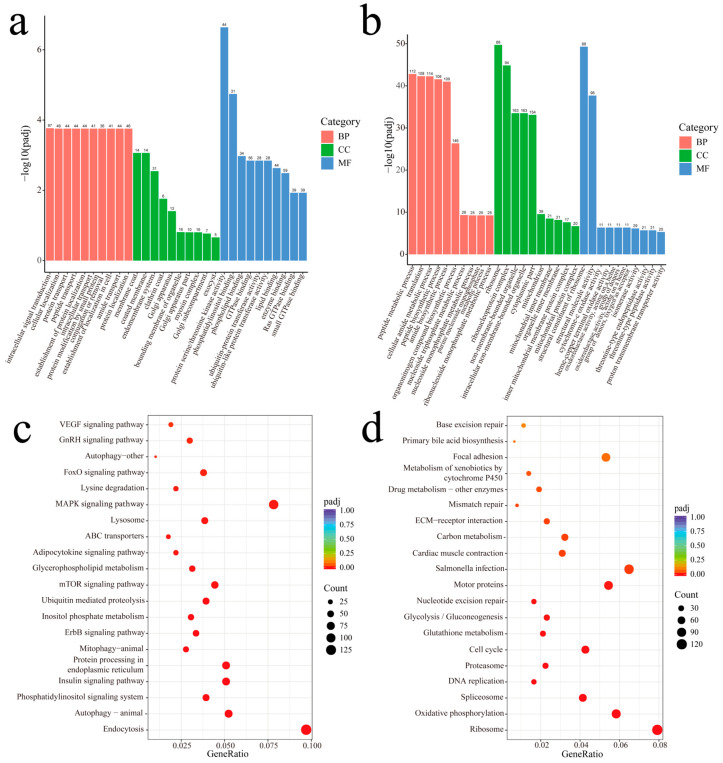
GO and KEGG enrichment analysis of up-regulated and down-regulated DEGs. (**a**) Enriched GO terms of up-regulated DEGs in P3 vs. P12 comparison. (**b**) Enriched GO terms of down-regulated DEGs in P3 vs. P12 comparison. (**c**) Enriched KEGG pathways of up-regulated DEGs in P3 vs. P12 comparison. (**d**) Enriched KEGG pathways of down-regulated DEGs in P3 vs. P12 comparison.

To explore the functions of these DEGs, we performed GO and KEGG enrichment analysis using up- and down-regulated genes, respectively. The up-regulated DEGs were enriched in 79 GO terms under the condition of *p-adj* < 0.05. The biological process (BP) most enriched terms were intracellular signal transduction, cellular localization, and protein transport, etc. Regarding the cellular components (CCs), the membrane coat, coated membrane, and endomembrane system, etc., were the most enriched. Further, protein serine/threonine kinase activity, phosphatidylinositol binding, and phospholipid binding, etc., were the GO terms most enriched in molecular function (MF) (Figure 6a). On the other hand, a total of 228 GO terms with *p-adj* < 0.05 were enriched in the down-regulated DEGs (Figure 6b). Terms related to BP included peptide metabolic process, translation, and cellular amide metabolic process, etc. In the CCs, a ribosome, ribonucleoprotein complex, and non-membrane-bounded organelle, etc., were observed. The structural constituent of the ribosome, structural molecule activity, and cytochrome-c oxidase activity, etc. were observed in MF. Interestingly, some BP categories related to cell culture in vitro, such as mitotic cell cycle, mitotic cell cycle process, cell cycle, etc., were also found significantly enriched (Table A1).

KEGG enrichment analysis found that 22 and 17 pathways were enriched in up-regulated and down-regulated DEGs, respectively (*p-adj* < 0.05). The KEGG pathways significantly enriched by up-regulated DEGs including Endocytosis, Autophagy-animal and the Phosphatidylinositol signaling system. Intriguingly, several of the KEGG pathways enriched were related to metabolism, for example, Inositol phosphate metabolism, Glycerophospholipid metabolism, and Lysosome (Figure 6c). On the other hand, the pathways enriched in down-regulated DEGs included Ribosome, Oxidative phosphorylation, and Spliceosome, etc. KEGGs associated with essential cellular processes such as cell division-related pathways (DNA replication, Cell cycle, Mismatch repair, and Nucleotide excision repair) and metabolism-related pathways (Glutathione metabolism, Glycolysis/Gluconeogenesis, Carbon metabolism, Drug metabolism-other enzymes, and the Metabolism of xenobiotics by cytochrome P450) were also found (Figure 6d).

### 2.6. Expression Level of KEGG Pathway Genes and qRT-PCR Validation

**Figure 7:**
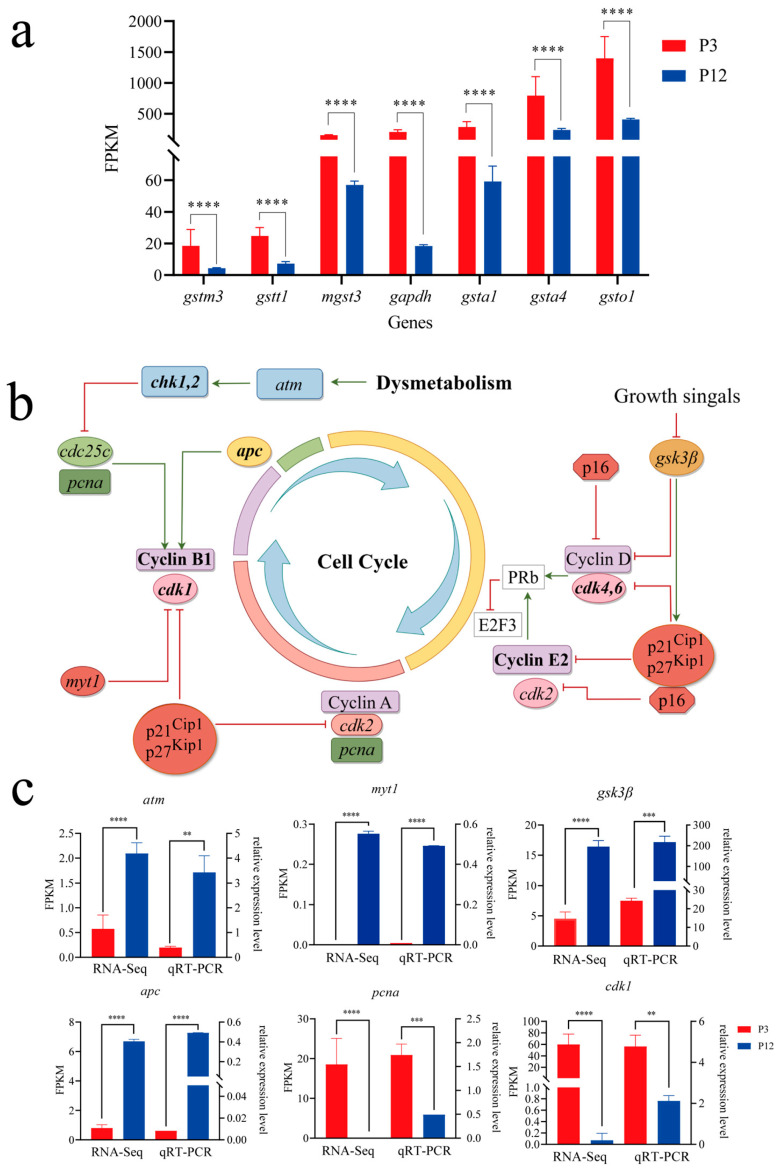
The signaling pathway and key genes involved in the cell cycle. (**a**) Expression level of metabolism-related genes in transcriptome data. (**b**) The schematic diagram of the cell cycle pathway. This pathway was drawn by figdraw. Positive regulation was indicated by green arrows, and negative regulation was indicated by red arrows. (**c**) The valid transcriptome assembly was verified by qRT-PCR (** *p* < 0.01; *** *p* < 0.001; **** *p* < 0.0001).

Based on the KEGG enrichment analysis, we observed that the expression of antioxidant-related genes, including glutathione S-transferase mu 3 (*gstm3*), glutathione S-transferase theta 1 (*gstt1*), microsomal glutathione S-transferase 3 (*mgst3*), glutathione S-transferase alpha 1 (*gsta1*), glutathione S-transferase alpha 4 (*gsta4*), and glutathione S-transferase omega 1 (*gsto1*), were significantly down-regulated in P12 compared to P3, indicating a reduced antioxidant capacity in P12 cells. Meanwhile, glyceraldehyde-3-phosphate dehydrogenase (*gapdh*), a commonly used metabolic marker, was significantly down-regulated in P12 compared to P3. The downregulation of these genes may indicate metabolic dysregulation associated with impaired cell function, which disrupted cell proliferation, survival, and overall metabolic homeostasis (Figure 7a, *p* < 0.0001).

In parallel, given the observed metabolic disorders arising from impaired cell function, we focused on several key genes related to the cell cycle. Compared to P3, Checkpoint Kinase 1 (*chk1*), Cyclin-dependent kinase regulator B1 (*ccnb1*), Cyclin-dependent kinase 1 (*cdk1*), E2F transcription factor 3 (*e2f3*), Cyclin-dependent kinase regulator E2 (*ccne2*), and Cyclin-dependent kinase 4 (*cdk4*) all significantly down-regulated in P12. A schematic diagram of the Cell Cycle pathway was presented in Figure 7b. The abnormal expression of these genes may indicate a disruption of the normal mitotic process, which may lead to halted cell growth and division. Based on the differentially expressed genes, GO/KEGG enrichment results, and relevant literature, we hypothesized that the abnormal cell division in vitro was caused by the metabolic disorder during cell culture.

The relative expression levels of key genes in the selected Cell Cycle pathway were validated by qRT-PCR using the same RNA samples, including Ataxia Telangiectasia Mutated (*atm*), Myelin Transcription Factor (*myt1*), glycogen synthase kinase 3 beta (*gsk3β*), Adenomatous Polyposis Coli (*apc*), proliferating cell nuclear antigen (*pcna*) and *cdk1*. Our data revealed that the expression changes in these six genes exhibited regulatory trends consistent with the transcriptome results (Figure 7c). These findings further supported the reliability of our transcriptome data.

## 3. Discussion

As an important marine economic fish, the value of the artificial cultivation of the *S. schlegelii* is increasing year by year. As a sex dimorphic species, the female fish grows about 25% faster than the male fish [1,3]. Therefore, the industry of *S. schlegelii* can be significantly improved by producing all-female seedings. The establishment of a germ cell line can provide a stable source of donors for the GCT of *S.schlegelii*, and realize the preparation of all-female offspring of *S.schlegelii*. In this study, for the first time, we continuously cultured *S. schlegelii* OSCs for 12 passages in vitro. At the same time, through RNA sequencing, we found that metabolic balance was crucial in sustainable cultured cells in vitro, and cell cycle regulation was key for cell division during cell culture.

Previous studies have indicated [19] that the ovarian cell line of *S. schlegelii* can be maintained in vitro up to the second passage. To optimize these culture conditions, we supplemented the complete culture medium with larval extraction and sterile serum derived from *S. schlegelii,* creating a culture environment that better resembled in vivo conditions. Using the method of single-cell isolation and culture, we successfully isolated and purified OSCs which could be sustained for 12 passages, retaining an oval morphology characteristic of stem cells [22].

In this study, SsOSCs were successfully expanded through 12 passages and exhibited several morphological changes, including an enlarged cell and nuclear size and a reduced nucleocytoplasmic ratio-hallmarks of cellular senescence during in vitro culture [23,24]. The P12 SsOSCs were notably larger than P3 cells, suggesting that morphological alterations occurred during long-term culture. Cytoplasmic enlargement has been found with in vitro aging in mammalian cells [25]. Moreover, the P12 cells showed a significantly reduced proliferative capacity compared to those from the primary culture (P3), which was a well-established feature of replicative senescence [26,27].The observed morphological changes in P12 SsOSCs were consistent with previous reports and support the conclusion that the cells underwent replicative senescence. Notably, despite signs of apoptosis, the continued expression of stem cell-related genes indicated that the P12 cells retained their identity as undifferentiated OSCs.

It is well documented that *ddx4* and *cdh1* serve as important germ cell markers [28,29,30,31]. Additionally, *klf4* is recognized as a pluripotency marker [14]. In contrast, *sox9* and *fshr* are generally considered somatic cell markers [32]. Genes such as *nanos2* [33] and *foxl2* [34] are specifically overexpressed in ovarian granulosa cells. These expression patterns suggested that SsOSCs likely originate from germ stem cells rather than somatic ovarian cells. Alkaline phosphatase activity, highly expressed in embryonic germ and stem cells [35,36], was also detected in SsOSCs, indicating the retention of stem cell properties after multiple passages in vitro. Karyotype analysis showed a diploid chromosome number of 2n = 48, consistent with previous reports for this species [37].

We demonstrated that OSCs from *S. schlegelii* could be cultured for 12 passages, which was the longest reported duration for marine fish OSCs. These cells showed typical stem cell characteristics during early passages, including a high alkaline phosphatase activity, stable diploid karyotype, and expression of germline markers. However, with continuous culture, the cells gradually exhibited senescence phenotypes, including reduced proliferation, increased apoptosis, and transcriptomic changes indicating stress.

Compared to P3 cells, the expression of *mgst3*, *gstm3*, *gapdh*, *gsta1*, *gsta4*, *gstt1*, and *gsto1* were significantly down-regulated in the P12 cells, indicating impaired cellular function. *mgst3* and *gstm3* could facilitate glutathione binding to toxic electrophiles, protecting cells from oxidative stress and damage [38,39]. Similarly, *gsta1* and *gsta4* detoxify reactive oxygen species (ROS) and lipid peroxidation products [40,41]. *gstt1* and *gsto1* mediate glutathione’s conjugation to various harmful substrates, including carcinogens and drugs [42,43]. *gapdh* is a key glycolytic enzyme linking metabolism to energy production and redox balance [44]. Downregulation of these genes suggests reduced antioxidant capacity and compromised oxidative stress management, leading to the accumulation of ROS and cellular damage. Impaired detoxification may lead to the buildup of toxic metabolites, disrupted homeostasis, and negative effects on cell proliferation, survival, and differentiation, ultimately weakening the health and function of cultured OSCs.

Cellular metabolic imbalance disrupted the cell cycle. Analysis of transcriptome data revealed aberrant cell cycle-related gene expression. Cyclin-dependent kinases (CDKs) and their cyclin partners orchestrate cell cycle transitions and stem cell fate [45]. Inactivation of the *gsk3β*-Cyclin D1 axis arrests cells in the G0/G1 phase [46], while *cdk4/6* and *cdk2*-Cyclin E complexes promote Rb phosphorylation and facilitate *E2F*-driven DNA replication [47,48,49,50]. *pcna* supports DNA synthesis during the S phase [51], and *myt1* inhibits *cdk1* to regulate G2/M transition [52]. Furthermore, *atm* activation or *apc* suppression can trigger Cyclin B1’s degradation and mitotic exit [53,54,55]. Several of these genes, including *atm*, *myt1*, *gsk3β*, *apc*, and *pcna,* were validated by qPCR and showed expression patterns consistent with transcriptomic data, supporting their role in OSC senescence.

In summary, we established a culture system in vitro for OSCs from *S. schlegelii* and successfully maintained them for 12 passages. Comparative transcriptomic analysis between P3 and P12 cells revealed that cell cycle arrest was a key bottleneck limiting prolonged culture, while metabolic integrity was essential for sustaining cellular proliferation and viability. We identified key glycolysis-related gene (*gapdh*), glutathione metabolism genes (*gstm3*, *gsto1*, *gsta1*, *gsta4*, *gstt1*, *mgst3*), and cell cycle regulators (*cdk1*, *atm*, *myt1*, *pcna*, *gsk3β*, *apc*) whose downregulation may underlie the observed senescence. These findings indicated a potential link between metabolic homeostasis and cell cycle progression in regulating the proliferative of germline stem cells during long-term in vitro culture. While further validation is needed, the *S. schlegelii* OSC culture system provides a promising model for exploring the cellular mechanisms underlying stem cell senescence. This work also lays the groundwork for future strategies aimed at enhancing reproductive biotechnology. For breeding, OSCs enable the rapid generation of genetically improved fish via GCT [36]. Molecular insights optimize culture systems may enhance stem cell performance. Notably, OSCs possess substantial untapped potential awaiting further exploration.

## 4. Materials and Methods

### 4.1. Fish and Reagent Preparation

One-year-old *Sebastes schlegelii* individuals (*n* = 6, body weight 90.5 ± 12.0 g, total length 17.8 ± 2.1 cm) were obtained from the Muping Aquaculture Base (Yantai, Shandong Province, China). Prior to the experiments, the fish were acclimated for 2 weeks in 500 L circular tanks with flow-through seawater under natural photoperiod conditions. During acclimation, fish were fed twice daily with commercial pellets and showed normal feeding behavior and no signs of disease.

Blood was collected from healthy individuals using sterile syringes and transferred into centrifuge tubes. After standing at 4 °C for 2 h, the blood was centrifuged at 8000 rpm for 15 min at 4 °C. The supernatant was filtered through a 0.22 μm sterile filter to obtain cell-free serum. The complete culture medium were consisted of Leibovitz’s L-15 medium (Solarbio, Beijing, China), supplemented with 20% fetal bovine serum (FBS, Gibco, Billings, MT, USA), 100 IU/mL penicillin, 0.1 mg/mL streptomycin, 50 μg/mL gentamicin (Solarbio, Beijing, China), 1% *S. schlegelii* serum, 1% *S. schlegelii* larval extraction, 10 mmol/mL HEPES (Gibco, Billings, MT, USA), 0.34 μL/100 mL 2-mercaptoethanol (Macklin, Shanghai, China), 2 ng/mL recombinant human basic fibroblast growth factor (bFGF, Beyotime, Jiangsu, China), and 2 ng/mL recombinant human leukemia inhibitory factor (LIF, Beyotime, Jiangsu, China).

### 4.2. Primary Cultivation of Oogonial Stem Cells from S. schlegelii

Healthy female *S. schlegelii* were selected and acclimated in the laboratory for 24 h. The fish were anesthetized in seawater containing 200 mg/L Tricaine methanesulfonate (MS-222; Sigma, Shanghai, China), followed by surface sterilization using 75% ethanol. Dissection was conducted under sterile conditions. Ovaries were excised, immediately transferred into L-15 medium containing penicillin–streptomycin and gentamicin, and rinsed 3–4 times to remove residual mesangial tissues and blood vessels.

The cleaned ovaries were minced into 1 mm^3^ fragments and digested with 0.25% trypsin-EDTA for 10–15 min at room temperature (RT, 25 ± 1 °C). The resulting cell suspension was filtered through a 40 μm mesh to obtain single cells, which were seeded into 25 cm^2^ cell culture flasks and maintained at 24 °C. During primary culture, half of the medium was refreshed every 5–7 days, and cell attachment and morphology were monitored continuously.

Once OSC-like cells appeared, putative SsOSCs were transferred sequentially from 96-well plates to 48-, 24-, 12-, and 6-well plates and eventually to cell culture flasks for expansion. When the cultures reached 80–90% confluency, the cells were passaged. For subculturing, the spent medium was removed, and the cells were rinsed with L-15 containing antibiotics and digested with 1 mL of 0.25% trypsin-EDTA. Once most of the cells had rounded up, they were split at a 1:1 ratio into new flasks with fresh medium. This procedure was used for all subsequent passages.

### 4.3. Chromosome Karyotype Analysis

The 10th passage SsOSCs were cultured in 75 cm^2^ flasks at 24 °C for 24 h, then treated with 0.25 μg/mL colchicine for 2 h. After washing with PBS, the cells were digested by trypsin, subjected to hypotonic treatment (0.075 M KCl, 37 °C, 40 min), and fixed in Karnaugh’s solution. Following repeated fixation cycles (3–4×, 40 min each), the cells were dropped onto chilled slides, Giemsa-stained, and imaged by brightfield microscopy.

### 4.4. Alkaline Phosphatase Staining

The 10th passage SsOSCs with 90% confluence were transferred to 6-well plates for culturing. Alkaline Phosphatase activity experiments were performed with a Leukocyte Alkaline Phosphatase Kit (Beyotime, Jiangsu, China) according to the manufacturer’s instructions. After that, the cells were transferred to an incubator and cultured in the dark for 12–24 h. The cells were observed and photographed under an inverted microscope (IX73, OLYMPUS, Tokyo, Japan).

### 4.5. Total RNA Extraction, cDNA Reverse Transcription, and Polymerase Chain Reaction

Samples of P12 cells were collected, and the total RNA was extracted using TRIzol (Invitrogen, Waltham, MA, USA) according to the manufacturer’s instructions. The cDNA was synthesized using a Prime-Script™ RT Reagent Kit with gDNA Eraser (TaKaRa, Kusatsu, Japan) following the manufacturer’s manual.

Semi-qPCR was used to detect the expression of cellular marker genes in cells and gonads. The PCR system was as follows: DNA template 200 ng; upstream and downstream primers (10 µmol/L) 0.4 µL each; 2 × EasyTaq SuperMix 10 µL (Vazyme, Nanjing, China); RNA-free water to 20 µL. The PCR conditions were as follows: 94 °C predenaturation for 3 min; 35 cycles: denaturation at 94 °C for 15 s, renaturation at Tm for 15 s, and extension at 72 °C for 5 s; extension at 72 °C for 5 min; and storage at 4 °C. PCR products were analyzed by 1% agarose gel electrophoresis for specific bands. Primers were shown in Table A2.

### 4.6. Fluorescence in Situ Hybridization

OSCs were inoculated into 12-well plates (NEST, Wuxi, China) until cell fusion reached 80%. FISH fluorescent probes were synthesized using a multi-sequence hybrid probe by Servicebio Inc. (Servicebio, Wuhan, China). Fluorescence in situ hybridization was performed with a FISH kit (Servicebio, Wuhan, China) according to the manufacturer’s requirements. Images were acquired and analyzed using inverted fluorescent microscope (IX73, OLYMPUS, Tokyo, Japan). The probe sequences were shown in Table A2.

### 4.7. Transcriptome Library Construction and Sequencing

For transcriptome sequencing, total RNA was extracted from passage 3 (P3) and passage 12 (P12) cells using TRIzol (Invitrogen, Waltham, MA, USA). RNA integrity was evaluated using an Agilent 5400 (Agilent Technologies, Santa Clara, CA, USA). All samples exhibited RIN values ≥ 6.7 (mean = 7.6, *n* = 6). PolyA + mRNA was extracted, fragmented, and reverse-transcribed into cDNA. After adapter ligation and size selection (370–420 bp), the libraries were PCR-amplified. This study employed the Illumina NovaSeq X Plus sequencing platform (Illumina, San Diego, CA, USA) for high-throughput sequencing, utilizing a paired-end 150 bp (PE150) sequencing strategy.

### 4.8. Data Filtering and Genome Mapping

Raw sequencing data were processed using SOAPnuke (v1.4.0) [56] and Trimmomatic (v0.36) [57] to remove adapters, exclude reads with >3% ambiguous bases, and discard sequences with >20% low-quality bases (Q ≤ 5). High-quality reads were aligned to the *S. schlegelii* genome (GCA014673565.1) via HISAT2 (v2.0.5) [58] and Bowtie2 (v2.2.5) [59] using default parameters. The feature Counts (1.5.0-p3) was used to calculate the readings mapped to each gene, the Fragments Per Kilobase of exon model per Million mapped fragments (FPKM) of each gene based on its length, and the readings mapped to that gene. DESeq2 software (1.20.0) was used to screen differential expression genes (DEGs). The unigenes with a *p-adjust* < 0.05 and |log2(fold-change)| ≥ 1 were identified as significant DEGs.

### 4.9. Identification of Gene Ontology and Kyoto Encyclopedia of Genes and Genomes Enrichment Analysis

The functional annotation of DEGs was performed using the cluster Profiler (v3.8.1), incorporating gene length bias correction. Statistically significant enrichment was defined as a false discovery rate (FDR)-adjusted *p-adjust* < 0.05 for both GO terms and KEGG pathways.

### 4.10. Evaluation of Gene Expression by qRT-PCR

To further validate the cell apoptosis and the reliability of the RNA-seq data, apoptosis-related genes and cell cycle-related DEGs were selected for qRT-PCR verification. Primers were designed using Primer 5.0 and synthesized by Qingdao Ruibo Biotechnology Co., LTD., Qingdao, China, (Table A2). qRT-PCR was performed with the QuantiNova SYBR Green PCR Kit (Qiagen, Hilden, Germany). The methods and reaction systems were in accordance with the instructions. The reaction procedure was performed on a LightCycler^®^ 480 II system (Basel, Switzerland) as follows: 95 °C for 2 min; 40 cycles of 95 °C for 5 s and 60 °C for 10 s; 95 °C for 15 s, 60 °C for 1 min, +1 °C/min; and 95 °C for 15 s. *β-actin* was used as an internal reference gene. Three replicates of each reaction were included in this study, and the relative expression of the genes was analyzed using the 2^−∆∆CT^ method.

All data are represented by mean stand error (x ± SEM). Differences between groups were analyzed by *t*-test using GraphPad Prism 9.5.0 software (GraphPad, San Diego, CA, USA). All differences were considered statistically significant when *p* < 0.05.

## Figures and Tables

**Table 1:** Summary of transcriptome sequencing.

Samples Name	Read Length	Raw Reads	Clean Reads	Q20 (%)	Q30 (%)	Mapping Ratio (%)
P3_1	150	42,345,470	42,257,282	98.44	95.72	76.47
P3_2	150	46,279,568	41,974,512	98.55	95.98	77.12
P3_3	150	49,217,418	45,079,374	98.34	95.48	77.64
P12_1	150	46,206,448	42,474,044	98.27	95.24	76.02
P12_2	150	46,206,448	42,961,838	98.55	95.98	74.95
P12_3	150	48,191,322	48,113,248	98.6	96.09	74.63

## Data Availability

The data are contained within the article. RNA-seq data are submitted in NCBI (BioProject: PRJNA1132777).

## Supplementary Materials

The following supporting information can be downloaded at: https://www.mdpi.com/article/10.3390/ijms26146772/s1.

## Appendix A

**Table A1:** GO terms related to cell cycle enriched by down-regulated DEGs.

Category	GOID	Description	GeneRatio	BgRatio	Pvalue	Padj	Count
BP	GO:0000278	mitotic cell cycle	11/1,150	17/7,527	5.14 × 10^−6^	6.42 × 10^−5^	11
BP	GO:1903047	mitotic cell cycle process	9/1,150	14/7,527	4.25 × 10^−5^	0.000403269	9
BP	GO:0007049	cell cycle	19/1,150	56/7,527	0.000404622	0.002917837	19
BP	GO:0140014	mitotic nuclear division	6/1,150	10/7,527	0.001514911	0.00923374	6
BP	GO:0022402	cell cycle process	12/1,150	37/7,527	0.006953496	0.028944634	12

**Table A2:** Primers and probes for gene expression analysis (semi-qPCR, qRT-PCR) and FISH.

Genes	Sequences (5′-3′)	Product Length	Tm	Usage
*β-actin*-F	AAGGACCTGTACGCCAACAC	248 bp	57.5 °C	Semi-qPCR and qRT-PCR
*β-actin*-R	TTCCTGTGGACAATGCTGGG			
*nanos2*-F	CTTCGACATGTGGCACGACT	314 bp	55.5 °C	Semi-qPCR
*nanos2*-R	CGTCTGATTTCAGCCGGTGT			
*foxl2*-F	CAACACCACGACCAAGGAGA	364 bp	57.5 °C	
*foxl2*-R	TCGTCTCCGGTAGTTTCCCT			
*ddx4*-F	GCAGAACAGATAGCAGTGAAT	550 bp	55.5 °C	
*ddx4*-R	TGCTGCAGAATAGGGAGCAGG			
*sox9*-F	CAACGCGGAACTCAGCAAAA	367 bp	55.5 °C	
*sox9*-R	CTCACGCTTCAGGTCAGGTT			
*fshr-F*	CTCGACCGTAAACTTCGCCT	247 bp	59.0 °C	
*fshr-R*	GGTTGTGGACGGTCAGGTAG			
*cdh1*-F	CCGTCCTGGCTAAAGTGTGT	418 bp	55.5 °C	
*cdh1*-R	TTGTCGGCTGCCTTCAGATT			
*klf4*-F	CCCTGTGGAGGGGTCTACTT	311 bp	60.0 °C	
*klf4*-R	TTCATATGGAGAGCAAGGTGGT			
*ddx4*	CGGCTTCACATAACCCGACTTGCT	\	\	FISH
	TCCAGGACCGCTGAACGCA			
*klf4*	GGCTGCGAGATGGTGCAGGTACTG	\	\	
	GGCTTCGGTTCCTCCGGTAAGCAG			
	TCCCAGTCGCAGTGGTAAGGCTTC			
*p53*-F	TGCTGAGAGTGCAGTGTTGT	266 bp	59.9 °C	qRT-PCR
*p53*-R	GGATGGTCAGCACCCAGTAG			
*bax*-F	GGCGTGTGAAGGAAACAACA	245 bp	59.3 °C	
*bax*-R	AACAAGCCACCCCGTCTATC			
*bcl-2*-F	GAAGGAGATGAGTCCGCTGG	222 bp	59.9 °C	
*bcl-2*-R	TTTCTGGGCGATGAGCGAG			
*caspase-1*-F	AGAAACGTCCCACCAGAGGA	241 bp	60.5 °C	
*caspase-1*-R	CATCCTTACGGAGAGCAGCG			
*gsk3β*-F	AAGGTTCTGGGGACACCAAC	297 bp	59.7 °C	
*gsk3β*-R	GATAGACAGCTCGACGGGAC			
*cdk1*-F	GACCCGATGCTGGTCAAGAG	278 bp	60.3 °C	
*cdk1*-R	ATGGTCCCAGTGCTCCAAAC			
*atm*-F	GGTACGAGCCGCTTTAGTGA	292 bp	59.9 °C	
*atm*-R	AGGGAGCTTCATCCCTTCCT			
*apc*-F	CACTGACTCTTACCCGGCAG	269 bp	60.1 °C	
*apc*-R	GCTCTCTGTGATGCTGTCGT			
*pcna*-F	TCCACTCCTGCAAAGAGCAAA	216 bp	60.1 °C	
*pcna*-R	ATCGTCATCGTCAGCAGGCA			
*myt1*-F	GAGGGTCTCCTGAATGGCAC	300 bp	60.0 °C	
*myt1*-R	GTTGACCATGTCAGCCTCCA			
